# 

**Published:** 1920-04-17

**Authors:** 


### TUBERCULOSIS NOTES.

### Lay "Cures" for Consumption.

We see many wonderful things in the Sunday papers, and recently some space has been devoted to a consumption cure put forward by an uneducated layman. There are the usual testimonials from patients, all of them to the medical eye, or even to a health visitor's eye, cramful of possible fallacies. There is also the usual demand for investigation at a regular consumption hospital. Now, to refuse this looks, to the outsider, like prejudiced intolerance, and yet there are ever so many good reasons for such an attitude. In medicine the layman can have no historical sense; he cannot know that for at least the last forty years there has been a new specific remedy at least every three months, and that each one has gone down the stream of time. The proper answer to such a demand is a hearty affirmative, with one single proviso: let such remedy first endure the formidable test of time; then, if its unadvertised and uncommercial use is greater at, say, the end of three years than at the beginning (which will surely be the case if it has any therapeutic virtue), a scientific investigation will at once be made. But cannot quack backers see for themselves that any real specific remedy for tuberculosis would succeed on its own merits, and even if violently opposed?

### Sanatoriums and Training Colonies.

Should these be combined or separate? The Ministry of Health says the former, but one local authority, at least, the latter. Which is right? The arguments for the first course include the important ones of economy. Precedent may also be quoted, for at the Treloar institution at Alton, Hants, a training college for crippled lads is combined on the same estate with the hospital for surgical tuberculosis, the one undertaking helping the other, since articles manufactured by those in the training college, nurses' outdoor cloaks, for example, are supplied to the hospital. All colonies for consumptives will make agricultural produce their staple output, and a sanatorium can take a great deal of this off their hands with the minimum of trouble and expense for carriage. Also, with the present dearth of medical men, a combined post, with its better emoluments, would be likely to be filled much sooner and better than two meagre openings. In the case of the Cheshire County Council, which, as we recently noted, has benefited considerably from the Red Cross funds, the argument of economy was granted, but it was stated by Alderman Raffles Bully that as regards Cheshire there were many objections to the combination. What these objections are he is not reported as saying; all the same we should like to hear them.

### Food for Consumptives.

A few months ago the Hanwell Urban Council called the attention of the Middlesex County Council to the necessity for supplying patients suffering from tuberculosis with milk at their homes. The County Council, however, came to the conclusion, as a result of the experience obtained by the tuberculosis officer, that on the whole it is not advisable to grant extras at home, as it tends to encourage the home treatment of patients in preference to allowing them to be sent to institutions. Moreover, it is not practicable to ensure that milk and other extras shall be used in the home for the patient only. The Hanwell Council has asked the County Council to reconsider the matter, especially in the interest of patients awaiting admission to institutions and other patients after discharge who require special diet. The Health Committee of the County Council has accordingly again gone into the matter, but while it recognises that advantage may accrue in securing to the use of patients awaiting admission to, and recently discharged from, institutions a supply of milk, it points out that several initial difficulties must be overcome before the County Council can carry out this work as a part of the treatment of tuberculosis, apart altogether from other questions of policy. The Committee has no means at its disposal of ensuring that milk supplied is used for the patient only and not in the general household, and invited the Hanwell Council to suggest suitable means which could be taken, either by the Council itself or otherwise, for securing control in the use of the milk. The Hanwell Council agrees that it is obviously impossible to ensure that the milk is used by the patient only. Taking all aspects of the case into consideration, the Middlesex Health Committee has declined to vary its previous decision.

### London Health Administration.

As has already been reported, the London County Council has prepared a scheme to co-ordinate the health services, and to enlist the co-operation of the various Borough Councils the County Council has had a conference with them.

As the St. Pancras Council reports, there are now two independent executive health authorities in each borough, namely, the County Council and the Borough Council. Thus overlapping and confusion are complained of.

The conference came to the conclusion that any redistribution of administrative functions between the L.C.C. and the Borough Councils should be as follows :—

London County Council—Legislation and co-ordination; making of by-laws, registration (other than that provided below), licensing; initiation of housing schemes (other than local), initiation of Parliamentary legislation; interchange of returns and information, special investigations; provision and maintenance of ambulances, institutions—hospitals, asylums, Poor-law infirmaries, casual wards, treatment centres, etc.; appeals from decisions of local authorities; and mandatory powers in the event of any default.

City and Borough Councils—All administrative (executive) functions in all branches of public-health work, including inspection and treatment of school children; insurance (other than institutional); Poor-law medical service (other than institutional); registration of births, deaths, and marriages; vaccination; lunacy; venereal diseases, etc.

The conference appointed a Committee to go further into the matter.

### HOSPITAL RETURNS FOR 1919.

### A Widespread Story of Heavy Deficits.

Norfolk and Norwich Hospital.—Of the income from all sources, £30,379, the sum of £10,552 was received for the treatment of soldiers and discharged men. The expenditure amounted to £40,981, and the overdraft at the bank at the end of the year was £14,245.

Essex County Hospital, Leicester.—The accounts show a deficit of £3,687 on the year's working, although there are large increases under every heading of income, subscriptions being increased by £542, and donations £3,555 higher than in 1918.

The Royal Maternity Charity.—The income for the year was £1,385 and the expenditure £895. The bank overdraft has been reduced to £2,555.

St. George's Hospital.—The total income (including £11,820 from legacies) was £47,629, and the total expenditure £69,201.

The General Hospital, Birmingham.—The total income for 1919 was £48,485, and the total expenditure £66,381. Legacies, included in the figure mentioned, were £7,590.

Warneford General Hospital, Leamington Spa.—Total income, £12,826; total expenditure, £13,697. Under several headings, including surgery and dispensary and management, the accounts show a reduced cost per patient.

Cheltenham General Hospital.—The total income was £8,502, and the total expenditure £11,471. Legacies, which are not included above, amounted to £1,190.

Birmingham and Midland Hospital for Women.—The accounts do not readily show the hospital's position at the end of the year, but there was a deficit of £6,844 on the ordinary income and expenditure account. However, legacies amounting to £6,263 and special donations of £2,435 were received.

The Royal Chest Hospital.—Including £1,777 from soldier and other patients, and £2,882, grants towards the Tuberculosis Dispensary, the total income was £13,605. The total expenditure was £14,699.

Royal Southern Hospital, Liverpool.—Including £14,335 from legacies, the total income was £40,248 and the total expenditure £32,655. This made a surplus of £7,592, but there was a deficit of £4,398 in 1918.

British Hospital for Mothers and Babies, Woolwich.—The total income was £3,301, and the total expenditure £3,222. No legacies were received.

The Royal Hospital, Richmond.—£6,698 was received in income under all heads, and the hospital spent £8,136. There were no legacies. Collection-boxes brought in £1,751.

Gravesend Hospital.—Total income, £6,611; total expenditure, £8,710. Patients' payments amounted to £1,884.

Walsall General Hospital.—Legacies amounting to £4,550 were received, but only a small proportion of this amount is included in the income for the year, which is shown as £11,175. The total expenditure was £14,768.

The Royal Earlswood Institution.—The receipts and payments account show that £45,074 was received and £47,138 spent. The farm receipts, including supplies to the institution, are valued at £7,471, and the payments under the same account were £7,725.

Derbyshire Royal Infirmary.—The report is for the year ending September 28, 1919, and is very nicely printed and illustrated. The income has been well maintained during the war. Salaries and wages in 1919 were £2,000 higher than in 1918.

Great Yarmouth Hospital.—The financial year ends in May, and the annual report is issued later in the year. The accounts afford another instance of deferred expenditure on painting and renovations, which amount to over £1,000. Fortunately a surplus of income over ordinary expenditure met this extra expense.

Liverpool Maternity Hospital.—There are only twenty-three beds and three camp-bedsteads available in the hospital, but the average daily occupation was twenty-three. The Board have noticed the low standard of education of a large number of women desirous of becoming midwives. Many of them can barely read and write.

West Suffolk General Hospital, Bury St. Edmunds.—The accounts are not kept on the Uniform System, but we understand will be so kept from 1920. The receipts amounted to £7,168 and the payments to £7,569. Endowments produce a revenue of £2,099. The report, which is complete, was one of the earliest received.

